# MOFF2: A Transferable Coarse-Grained Protein Force Field for Predictive Condensate Simulations

**DOI:** 10.64898/2026.06.10.731384

**Published:** 2026-06-10

**Authors:** Shuming Liu, Yumeng Zhang, Ivan Riveros, Cong Wang, Bin Zhang

**Affiliations:** † Department of Chemistry, Massachusetts Institute of Technology, Cambridge, MA 02139, USA

## Abstract

Coarse-grained protein force fields enable simulations of biomolecular systems at length and time scales that are difficult to access with atomistic models, but achieving transferability across folded, intrinsically disordered, and multidomain proteins remains challenging. A central difficulty is that one-bead-per-residue models must represent chemically specific residue interactions while also absorbing solvent-mediated and many-body effects into a simplified energy function. Here, we present MOFF2, a transferable coarse-grained protein force field that combines residue-pair-specific interactions with a density-dependent many-body potential. MOFF2 is optimized using a two-stage strategy: bottom-up parameter learning from heterogeneous reference ensembles followed by refinement against experimental conformational observables. The resulting model provides balanced performance across ordered proteins, intrinsically disordered proteins, and multidomain proteins, and predicts condensate saturation-concentration trends for A1-LCD variant systems. Analysis of the learned parameters reveals chemically interpretable interaction patterns and density-dependent effects that explain the model’s improved transferability. These results demonstrate that combining a generalized coarse-grained energy function with data-driven optimization can produce a practical and interpretable force field for protein conformational and condensate simulations.

## Introduction

Biomolecular condensates have emerged as a fundamental mechanism for organizing cellular biochemistry. ^1-5^ These dynamic, membraneless assemblies regulate diverse processes, including transcription, signaling, and genome organization. Their ability to concentrate specific biomolecules within distinct chemical environments enables precise spatial and temporal control of cellular reactions. ^1,4,6,7^ Dysregulation of condensate formation has been linked to neurodegenerative diseases and cancer, motivating intensive efforts to understand how amino acid sequences encode condensate stability, internal organization, and material properties, often described as the molecular grammar of phase separation. ^4,8-39^ Decoding this molecular grammar represents a central challenge in connecting sequence-level interactions to emergent mesoscale behaviors.

Molecular simulations offer a powerful means to address this challenge. ^11,22,29,40-46^ They provide atomistic to mesoscopic resolution of biomolecular assemblies and enable controlled perturbations of sequence and interaction strength to reveal the determinants of condensate formation. Coarse-grained (CG) models, which reduce the system representation while retaining residue-level chemical specificity, are particularly well suited for exploring condensate organization and thermodynamics at biologically relevant scales. ^11,23,25-28,34,39,42,43,47-54^ Among these, one-bead-per-amino-acid (1BPA) models have yielded key mechanistic insights into molecular interactions that drive phase separation. ^23-27,47,48,50,51^

CG models, however, often compromise on accuracy. They typically adopt pairwise additive interactions, analogous to those used in explicit-solvent force fields, to describe residue-residue interactions. Different parameterization strategies have been introduced, ranging from empirical mixing rules based on hydrophobicity scales to potentials of mean force derived from atomistic simulations or optimization against experimental observables. ^55-62^ Despite these efforts, it is well established that coarse-graining introduces many-body effects ^55,63-65^ that cannot be faithfully represented by pairwise interactions alone.

The compromise in accuracy of CG models is reflected in their lack of transferability. Several studies have shown that while 1BPA models can reproduce the dimensions of intrinsically disordered proteins (IDPs), often quantified by the radius of gyration (*R_g_*), they generally fail to stabilize folded proteins or to capture realistic conformations of multidomain systems. ^22,30,56,62,66-68^ Although folded domains are frequently constrained in simulations, an accurate description of their underlying energetics remains essential for maintaining the correct thermodynamic balance between folded, disordered, and associated states. Misrepresenting these interactions can distort the coupling between ordered and disordered regions, alter interdomain packing, and bias condensate thermodynamics. To address such deficiencies, some models employ different representations or interaction potentials for folded and disordered domains. ^51,69^ Although these artificial treatments can improve specific cases, they lack rigorous physical justification and may reduce model generality when applied to new systems. These limitations underscore the need for a physically grounded, transferable framework capable of describing folded, disordered, and hybrid proteins within a single potential.

Here we present **MOFF2**, a general protein CG force field designed to achieve consistent accuracy across folded, disordered, and multidomain systems. To address the limitations of pairwise additive models, MOFF2 incorporates a density-dependent many-body potential inspired by the AWSEM force field ^55^ that captures local packing and solvent-mediated effects arising from coarse-graining. In parallel, we developed a data-driven optimization framework that enables efficient parameterization of complex energy functions using large and heterogeneous training datasets. The force field is trained against conformational ensembles and experimental observables spanning ordered proteins (OPs), IDPs, and multidomain proteins (MDPs), thereby ensuring balanced performance across diverse structural contexts.

We demonstrate that MOFF2 reproduces single-chain conformational statistics across protein classes with high fidelity. The model also quantitatively captures experimental trends in condensate saturation concentrations (*C*_sat_). By unifying the treatment of folded and disordered proteins within a single physically grounded framework, MOFF2 provides a transferable platform for simulating protein conformations and phase behavior.

## Results

### MOFF2: Model Overview

The limited transferability of existing 1BPA models remains a major obstacle to predictive simulations of biomolecular condensates. This limitation arises in part from the simplified interaction potentials commonly employed in these models, which often rely on pairwise additive interactions and therefore provide only a limited description of the physical factors governing protein association and conformational stability. ^11,23,25-28,42,43,47-53,70^

**Figure 1:**
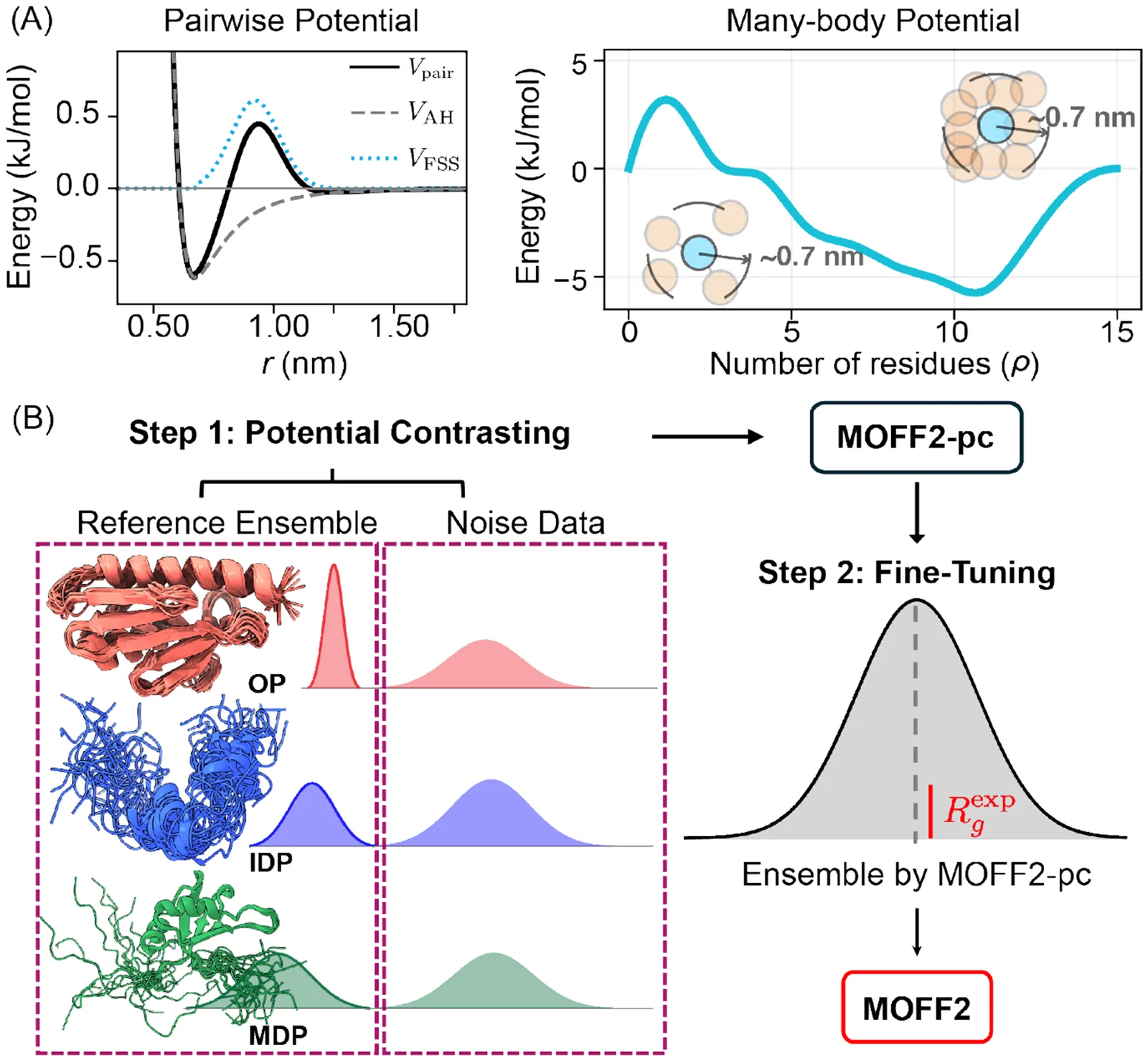
MOFF2 energy function and optimization workflow. (A) MOFF2 extends conventional 1BPA models through a flexible energy function that includes bonded and electrostatic interactions together with three nonbonded components: residue-specific pair interactions based on the Ashbaugh-Hatch (AH) potential (V_AH_), ^71^ a Gaussian first-solvation-shell correction (*V*_FSS_) that accounts for desolvation barriers during contact formation, and a density-dependent many-body term that captures environment-dependent interactions. (B) Two-stage optimization framework combining bottom-up learning from conformational ensembles and top-down refinement against experimental measurements.

To address these limitations, we developed MOFF2, a next-generation CG force field that extends conventional 1BPA models through a more expressive energy function. In addition to residue-specific pair interactions and screened electrostatics, MOFF2 incorporates both first-solvation-shell corrections and density-dependent many-body interactions (Figure 1A). Together, these terms enable the model to account for hydration effects, desolvation barriers, and environment-dependent packing contributions that are difficult to represent within conventional pairwise formulations.

The pairwise interaction term in MOFF2 includes both direct amino-acid contacts ^71^ and an explicit first-solvation-shell correction that captures the energetic barrier associated with displacing solvent molecules during contact formation. As a result, residue interactions are determined not only by their contact stability but also by the free-energy cost of removing intervening solvent. This additional level of physical realism improves the description of protein cores and intermolecular interfaces, where hydration effects play a critical role. ^66,72,73^

To further account for collective effects arising from coarse-graining, MOFF2 includes a density-dependent many-body potential. Unlike pairwise interactions, the many-body term introduces an explicit dependence on the local packing density surrounding each amino acid. This allows the model to distinguish between physical environments that would otherwise appear identical in a pairwise description. For example, amino acids buried within a densely packed protein core experience different effective interactions than those in expanded or dilute conformations. ^65,74-78^ By accounting for these differences, the many-body potential captures important effects such as hydrophobic burial, solvent-mediated stabilization, and local packing cooperativity, thereby enabling a unified description of OPs, IDPs, and MDPs.

### Transferability Across Protein Classes

The richer functional form of MOFF2 introduces a larger number of parameters and therefore requires a systematic optimization strategy. To address this challenge, we employ a two-stage framework that combines complementary bottom-up and top-down approaches (Figure 1B). First, the force field is trained against diverse conformational ensembles spanning OPs, IDPs, and MDPs. This stage uses potential contrasting optimization ^79,80^ to reproduce the structural distributions of the reference ensembles. Second, the resulting model is refined against experimental observables using a thermodynamic reweighting procedure. ^81,82^ Together, these steps enable efficient parameterization of a transferable force field while integrating information from both high-resolution simulations and experiments.

**Figure 2:**
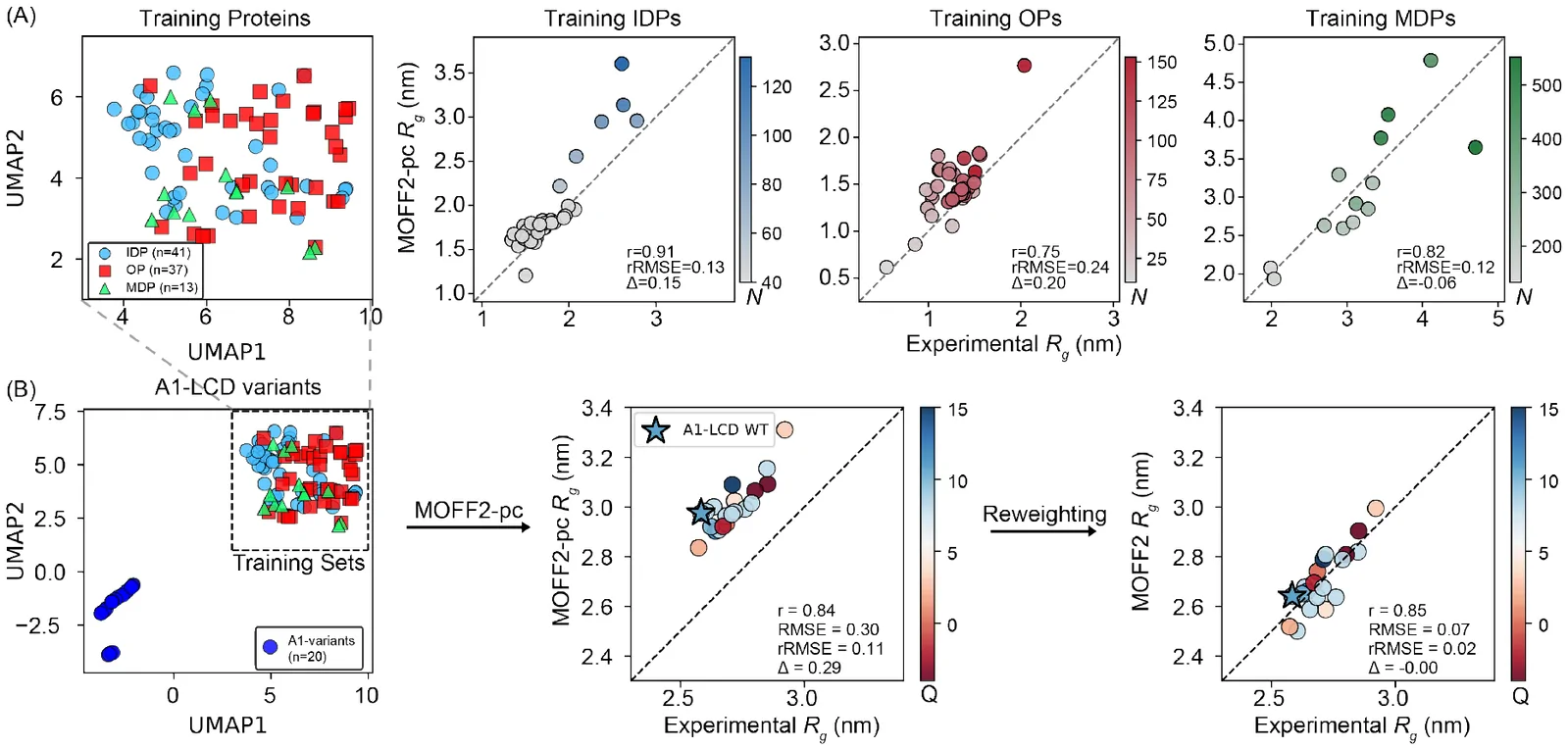
Two-stage optimization of MOFF2 establishes transferability across protein classes and sequence space. (A) Results of potential-contrasting optimization. The left panel shows a two-dimensional UMAP ^83^ projection of sequence-composition features for proteins used for model optimization. The right panels compare reference and MOFF2-pc predicted ⟨*R_g_*⟩ values for proteins in the training set. Proteins are colored by chain length, and dashed lines indicate perfect alignment. Legends report the Pearson correlation coefficient (*r*), the relative root-mean-square error (rRMSE), and the mean signed deviation (∆) (see Methods section for detailed definitions). (B) Top-down refinement of sequence regions that are underrepresented during potential contrasting. The left panel shows the location of A1-LCD variants in the same sequence-feature space, highlighting their limited overlap with the potential contrasting training set. The middle and right panels compare simulation results from MOFF2-pc and MOFF2 with experimental values.

For the potential contrasting stage, the training set included 37 OPs, 41 IDPs, and 13 MDPs (Tables S1-S5), covering a broad range of chain lengths, amino-acid compositions, and structural organizations (Figure S1). The resulting model, MOFF2-pc, successfully reproduced the overall conformational trends observed across the training proteins. Strong correlations were obtained between predicted and reference ensemble properties (Figure 2A), indicating that the force field captures physical interactions that generalize across distinct structural classes.

Importantly, the various components of the MOFF2 energy function contributed synergistically to this performance. Models containing only pairwise interactions exhibited less balanced behavior between OPs and MDPs, whereas inclusion of the density-dependent many-body term improved transferability across protein classes (Figure S2). Introducing the first-solvation-shell correction further enhanced predictions for MDPs while maintaining accuracy for OPs and IDPs. Together, these results demonstrate that chemically specific pair interactions, local solvation effects, and many-body packing contributions are all required to achieve balanced performance.

To evaluate generalization beyond the training data, we next tested MOFF2-pc on an independent validation set consisting of 9 IDPs (Table S6) and 14 MDPs (Table S8) that were not included during optimization. The model retained strong predictive power for these proteins (Figure S3), indicating that the learned interactions provide a broadly transferable physical description. Nevertheless, a systematic deviation emerged for the A1-LCD variant family, where the model consistently overestimated chain expansion (Figure 2B). This behavior is consistent with the sequence-space distribution of the training set. A1-LCD variants are enriched in Gly and Ser residues and occupy a region of sequence space that is sparsely represented among the proteins used for potential contrasting (Figure 2B and Figure S1). These results suggest that while potential contrasting establishes a robust global model, additional refinement may be beneficial for sequence families that are underrepresented in the bottom-up training ensembles.

Rather than generating new atomistic reference ensembles for these proteins, which would be computationally demanding and may still suffer from incomplete convergence, we used MOFF2-pc as a physically meaningful starting point and refined it against experimental observables using a thermodynamic reweighting method. ^81,82^

For this refinement stage, we incorporated experimental measurements (⟨*R_g_*⟩) for 20 A1-LCD variants together with 14 MDPs and 21 OPs (Tables S7-S9). Including multiple protein classes prevents overfitting toward a single disordered protein family while preserving the global balance established during potential contrasting. The refinement substantially improved predictions for the A1-LCD variants and corrected the systematic overexpansion observed in the MOFF2-pc model (Figure 2B). At the same time, the accuracy for OPs and MDPs was maintained (Figure S4), indicating that the update improved local performance without compromising overall transferability.

**Figure 3:**
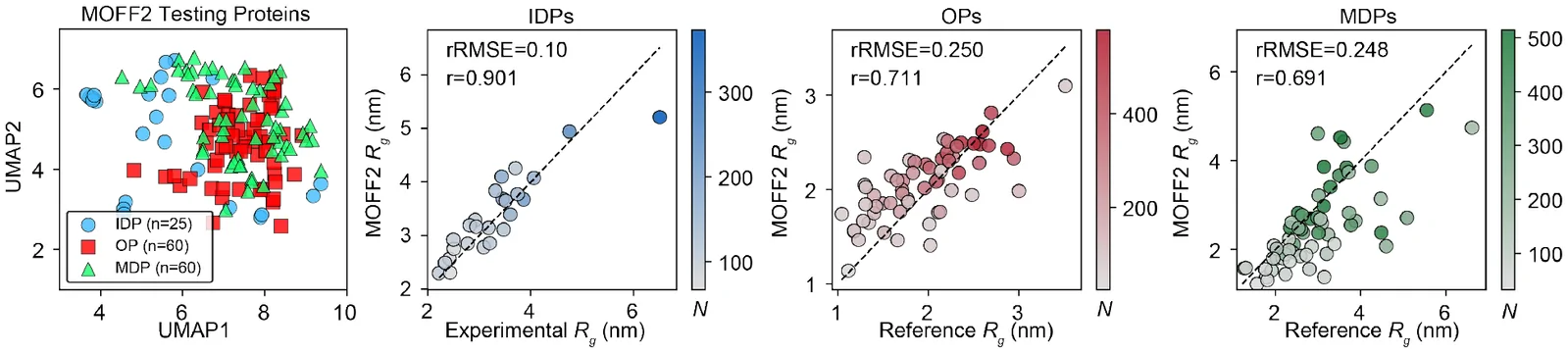
Evaluating MOFF2 on proteins unseen during training. The same plotting style as in Figure 2A is adopted here.

We refer to the resulting two-stage optimized force field as MOFF2. To further assess transferability, we evaluated MOFF2 on an independent test set of proteins that were not included in either the potential contrasting optimization or the reweighting refinement (Figure 3, Tables S10-S12). For IDPs, MOFF2 achieved strong agreement with experimental ⟨*R_g_*⟩ values. For OPs and MDPs, where comprehensive experimental conformational ensembles are generally unavailable, we used AlphaFold-predicted structures ^84^ as structural references. These references are expected to be more reliable for OPs than for MDPs because interdomain orientations and linker-mediated conformations are often predicted with lower confidence. Consequently, the larger deviations observed for some MDPs likely reflect uncertainties in the reference structures rather than deficiencies of the force field itself. Despite these challenges, MOFF2 preserved the overall conformational trends across all protein classes with strong correlations, supporting its transferability across OPs, IDPs, and MDPs.

### MOFF2 accurately predicts phase behavior of A1-LCD variants

Having established the accuracy of MOFF2 for single-chain properties, we next evaluated its ability to predict sequence-dependent liquid–liquid phase separation. A1-LCD variants provide a particularly stringent benchmark because extensive experimental measurements ^31,85-87^ are available for a diverse set of mutations that alter charge, aromatic composition, and sequence patterning, resulting in saturation concentrations that span a broad dynamic range.

**Figure 4:**
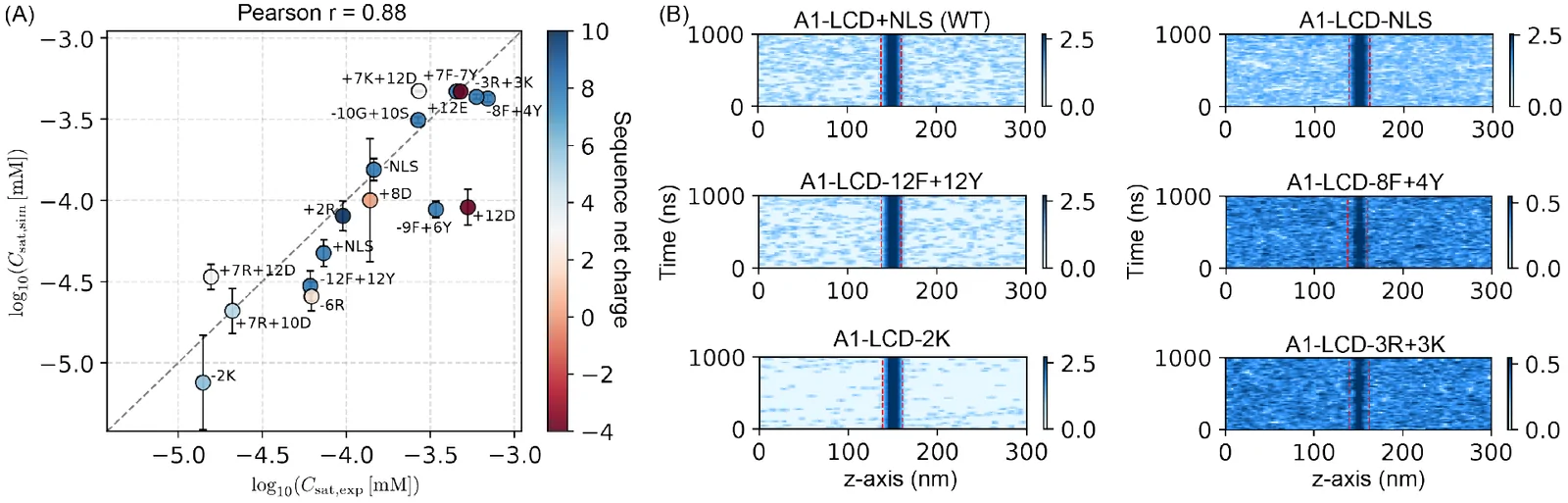
MOFF2 predicts sequence-dependent saturation concentrations of A1-LCD variants. (A) Comparison between simulated and experimental saturation concentrations, *C*_sat_, for A1-LCD variants. Both axes show log_10_(*C*_sat_) in mM. Proteins are colored by sequence net charge, and error bars indicate the standard error estimated from five-block averaging of the simulated *C*_sat_. (B) Representative slab-density profiles used to estimate *C*_sat_ from coexistence simulations (see Methods for details). Each heat map shows the protein concentration profile along the *z*-axis during the final 1 *μ*s of the trajectory that was used for analysis. Red dashed lines mark the separation between condensed and dilute phase used to estimate the *C*_sat_.

As shown in Figure 4A, MOFF2 reproduced the experimental saturation-concentration trends for most A1-LCD variants. ^86,87^ In particular, MOFF2 captured the effect of the nuclear-localization-signal (NLS) region disruption, distinguishing weaker phase separation ability for A1-LCD^-NLS^ relative to wild-type A1-LCD^+NLS^ (P105G/Y106S mutants). The predicted phase diagrams showed good agreement with in vitro measurements for variants spanning broad classes of sequence perturbations, including aromatic substitutions (A1-LCD^+7F-7Y^, A1-LCD^−12F+12Y^, A1-LCD^−8F+4Y^, and A1-LCD^−9F+6Y^), charge-pattern variants (A1-LCD^−3R+3K^), and acidic-residue mutations (A1-LCD^+12E^).

MOFF2 performed particularly well for several challenging charge-engineered variants. For example, the saturation concentrations of A1-LCD^+7R+12D^ and A1-LCD^−6R^ were predicted in close agreement with experiment. Likewise, MOFF2 substantially improved the description of highly charged variants, including A1-LCD^+8D^, A1-LCD^+12E^, and A1-LCD^+12D^, for which Mpipi-Recharged, among the most accurate 1BPA models for LLPS prediction, still underestimates the saturation concentration by approximately two orders of magnitude. ^88^

The largest discrepancies were observed for Trp-containing variants, including A1-LCD^allW^ A1-LCD^FtoW^, A1-LCD^YtoW^, and A1-LCD^W-^ (Table S13). This limitation is consistent with the relatively sparse representation of Trp-rich IDP sequence environments in the current training datasets (Figure S1). Consequently, these variants highlight a specific region of sequence space where the present parameterization remains weakly constrained, rather than indicating a general failure to predict A1-LCD phase behavior.

To place these results in context, we compared MOFF2 directly with Mpipi-Recharged on the same A1-LCD benchmark set (Table S13). The overall deviation was calculated as the mean absolute difference between simulated and experimental $C_{\text{sat}}$ values in units of M, $\langle |C_{\text{sat}}^{\text{sim}}-C_{\text{sat}}^{\text{exp}}|\rangle$. Despite the larger errors associated with Trp-containing variants, MOFF2 achieved an overall deviation of 0.048, comparable to the value of 0.044 reported for Mpipi-Recharged. ^88^ These results demonstrate that MOFF2 achieves state-of-the-art accuracy for predicting sequence-dependent phase separation while retaining the broader transferability afforded by its physics-based parameterization.

### Learned pairwise and many-body interactions explain MOFF2 transferability

Finally, we analyzed the learned interaction parameters to understand how MOFF2 achieves balanced performance across protein classes and condensate-forming systems. Because MOFF2 decomposes residue interactions into direct pairwise contacts, first-solvation-shell corrections, and density-dependent many-body interactions, the model provides an opportunity to examine how distinct physical contributions emerge during optimization. We therefore asked whether these learned terms recover chemically interpretable features of protein biophysics and how they contribute to transferability.

**Figure 5:**
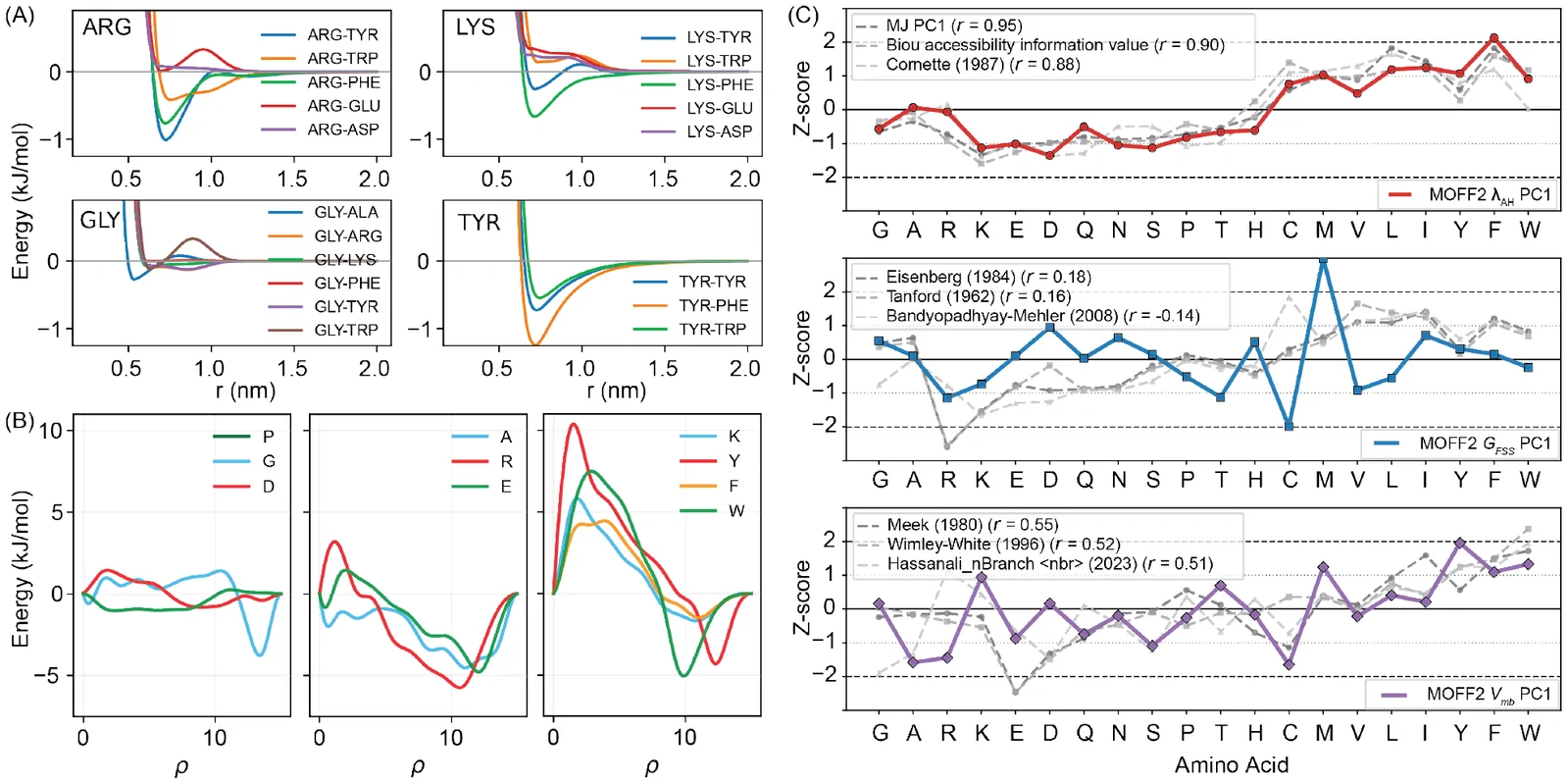
Interpretable chemical information from MOFF2 learned profiles. (A) Representative residue-pair interaction potentials learned by MOFF2. (B) Representative amino-acid-specific density-dependent many-body profiles. (C) Comparison between the first principal components (PC1) of the learned MOFF2 parameter sets: λ_AH_ (top), *G*_FSS_ (middle), and *V*_mb_ (bottom), and representative HPS references. Colored lines show the standardized MOFF2 PC1 profiles, gray lines show the three most correlated reference scales, and Pearson correlation coefficients (*r*) are reported in the legends.

We first examined the learned pairwise interaction strength λ_AH_, which describes the stability of direct residue–residue contacts. As shown in Figure 5A and Figures S5-S6, the learned interaction matrix captures several well-established sequence determinants of protein folding and phase separation. Most notably, MOFF2 naturally learns favorable cation–π interactions involving aromatic residues and positively charged side chains. Arg–aromatic contacts are substantially more favorable than the corresponding Lys–aromatic interactions, with Arg–Tyr among the strongest interactions in the matrix. This distinction is particularly important for condensate-forming proteins, where Arg-to-Lys substitutions frequently weaken phase separation despite preserving net charge. ^11,47,86,89,90^ MOFF2 also favors aromatic–aromatic interactions, including Tyr-containing contacts that have been repeatedly implicated in driving condensation of low-complexity domains. ^11,31,86,90-93^ Importantly, these chemically specific interaction patterns emerge directly from the optimization procedure and were not imposed through explicit interaction rules.

The first-solvation-shell correction *G*_FSS_ provides a complementary description of residue interactions. Whereas the pairwise term quantifies how favorable a contact is once formed, the solvation-shell term captures the energetic cost of transitioning between solvent-separated and direct-contact states. Several physically meaningful patterns emerge from the learned barriers. For example, Arg–Glu exhibits a pronounced barrier, reflecting the energetic cost of removing hydration waters before a salt bridge can form. ^94,95^ Interestingly, Gly–Trp also displays a substantial first-solvation-shell correction despite relatively weak direct-contact attraction. This feature may reflect the unique CH–π interaction between the glycine backbone and the Trp aromatic ring, which has recently been implicated in regulating phase separation of low-complexity domains. ^42,96-98^

The density-dependent many-body interactions provide a third and distinct layer of physical information. Rather than describing specific pairwise contacts, these terms encode how amino-acid preferences change with local packing density. As shown in Figure 5B and Figure S6C, aromatic and hydrophobic residues such as Trp, Tyr, and Phe become increasingly favorable in densely packed environments, consistent with burial-driven stabilization and the hydrophobic effect. In contrast, many polar and charged residues display weaker density dependence and remain comparatively favorable in dilute or partially packed environments. ^99-101^ The distinction between Arg and Lys is particularly notable. Arg exhibits favorable contributions across a broader range of densities, whereas Lys shows a stronger preference for highly packed environments. These observations suggest that the many-body term captures environmental preferences that cannot be represented through pairwise interactions alone and likely contributes to the transferability of MOFF2 across proteins spanning heterogeneous conformational space and interaction environments.

To place the learned interactions in the context of existing amino-acid descriptors, we compared the principal components of each MOFF2 interaction class (Figure S7) with established hydrophobicity and residue-interaction scales (Figures 5C and Figure S8). The direct pairwise contact term correlates strongly with the Miyazawa–Jernigan (MJ) ^102^ potential. This result is not unexpected because the pairwise contact parameters were initialized from the MJ matrix. Nevertheless, the high correlation indicates that the optimization procedure largely preserves the residue-contact preferences encoded in MJ while refining other components of the force field.

In contrast, the density-dependent many-body term shows strong correlations with interfacial hydrophobicity scales such as Wimley–White ^103^ (Pearson *r* = 0.52). This result is particularly notable because no such information was imposed during training, indicating that MOFF2 independently learned environmental preferences associated with the hydrophobic effect. The first-solvation-shell correction exhibits weaker correlations with existing scales, likely reflecting both its smaller magnitude and the absence of closely related descriptors in the chosen literature. Nevertheless, its strongest correspondence is with the Eisenberg hydrophobicity scale, ^104^ which measures residue interactions with water and is therefore conceptually aligned with the hydration-mediated processes captured by this term.

Together, the emergence of these chemically interpretable features provides a mechanistic explanation for the ability of MOFF2 to accurately describe various systems within a unified framework.

## Conclusions and Discussion

Here, we present MOFF2, a protein CG force field designed to capture residue-specific interactions, local solvation effects, and environment-dependent packing contributions within a unified framework. By coupling this physically motivated energy function with a two-stage optimization strategy that integrates potential contrasting and thermodynamic reweighting, MOFF2 establishes a transferable framework for protein modeling. The resulting model accurately reproduces conformational properties across OPs, IDPs, and MDPs while capturing sequence-dependent trends in biomolecular phase separation.

Despite these advances, the present study also highlights important opportunities for further improvement. Although the training set spans a broad range of proteins, the transferability of MOFF2 remains fundamentally constrained by the diversity of the available training data. Certain sequence environments, particularly aromatic-rich and low-complexity sequences, remain underrepresented. As illustrated by the residual deviations observed for several A1-LCD variants, interactions in these regions of sequence space are less tightly constrained than those represented more extensively in the training set. More generally, the current optimization strategy relies primarily on conformational ensembles and single-chain observables, whereas condensate thermodynamics are only indirectly incorporated through the underlying molecular interactions.

A promising direction for future development is therefore to extend the refinement framework directly to condensed-phase properties. The thermodynamic reweighting strategy introduced here is not limited to single-chain simulations and could, in principle, be generalized to condensate ensembles. Experimental observables such as saturation concentrations could then be incorporated directly into force-field optimization. Such an approach would enable systematic refinement of intermolecular interactions while preserving the physically meaningful baseline established through bottom-up learning.

More broadly, the combination of many-body interactions, atomistically informed training data, and statistically controlled optimization provides a general framework for developing next-generation CG force fields. These ideas should be readily extendable to nucleic acids, protein–nucleic acid assemblies, and other multicomponent biomolecular systems in which environment-dependent interactions play a central role. We anticipate that MOFF2 will serve both as a practical platform for studying biomolecular condensates and as a foundation for future efforts toward predictive CG modeling of complex cellular assemblies.

## Methods

### MOFF2 energy function

MOFF2 employs a 1BPA representation in which each amino acid is mapped to its C*α* atom. The resulting coarse-grained configuration is described by the energy function

(S1) $$V_{\text{MOFF}2}(r)=V_{\text{bond}}+V_{\text{elec}}+V_{\text{pair}}+V_{\text{mb}},$$

where $V_{\text{bond}}$ maintains chain connectivity, $V_{\text{elec}}$ describes screened electrostatic interactions, $V_{\text{pair}}$ describes residue-specific pair interactions, and $V_{\text{mb}}$ captures density-dependent many-body effects. Detailed mathematical forms are provided in the Supporting Information: *Explicit Energy Function.*

The pairwise nonbonded interaction accounts for both the direct amino acid contacts, described by the Ashbaugh–Hatch (AH) potential, ^105^ and the desolvation barrier, described by a Gaussian potential, that amino acids must overcome as they approach one another to form contacts.

To capture collective effects that are not representable by pairwise additive interactions, MOFF2 includes a density-dependent many-body potential,

(S2) $$V_{\text{mb}}=\sum_{i}S_{a_{i}}(\rho_{i}),$$

where $a_{i}$ is the amino-acid type, $\rho_{i}$ is the local residue density, and $S_{a_{i}}$ is an amino-acid-specific cubic B-spline function. ^106^

For proteins containing folded domains, an additional structure-based potential, *V*_native_, is used to maintain the reference fold. This term includes angle, dihedral, and native-contact restraints. During training and model evaluation of folded proteins, native contacts are restricted to contacts within the same continuous secondary-structure element, so long-range tertiary compaction is governed primarily by the transferable MOFF2 nonbonded terms.

### Model optimization

We employ a two-stage parameterization framework that integrates complementary bottom-up and top-down approaches. A brief overview is provided below, while detailed descriptions of the training data and optimization procedures can be found in the Supporting Information: *Force Field Optimization*.

#### Potential contrasting optimization

In the first stage, we parameterized MOFF2, using potential contrasting, ^79,80^ a variational framework that learns a coarse-grained energy function from reference configurational ensembles. Potential contrasting compares configurations sampled from the target data distribution with configurations sampled from a broader noise distribution. The optimized model assigns higher Boltzmann probability to data configurations than to noise configurations, thereby learning an energy landscape consistent with the reference ensemble without requiring force matching or repeated molecular dynamics simulations during optimization.

The optimized parameters include the 210 AH pair coefficients, the 210 Gaussian potential corrections, and the 240 coefficients of the density-dependent spline potential. Regularization terms were applied separately to the pairwise and density-dependent parameter classes to control parameter magnitude and improve transferability.

The training data include OPs, IDPs, and MDPs. For OPs and IDPs, configurations were collected from explicit-solvent atomistic molecular dynamics simulations. The OP training set includes long-timescale trajectories of 19 proteins reported by D. E. Shaw Research. ^107-109^ To increase sequence and structural diversity, we additionally generated trajectories for 18 ordered proteins using the a99SB-*disp* force field in OpenMM. ^110^ The IDP training set includes seven trajectories from D. E. Shaw Research ^108^ and 34 additional trajectories from Ref. 13.

For MDPs, converged explicit-solvent atomistic simulations are not practical because of their larger system sizes and slow interdomain rearrangements. We therefore generated coarse-grained conformational ensembles and reweighted them by a maximum-entropy procedure so that the reweighted ensembles reproduce experimental mean radii of gyration, ⟨*R_g_*⟩.

Noise ensembles were generated using HPS-Urry-based coarse-grained simulations. ^111^ For IDPs, umbrella sampling was performed along *R_g_*. For OPs, the HPS-Urry potential was supplemented with *V*_native_, and umbrella sampling was performed along the root-mean-square deviation (RMSD) from a representative folded reference structure. Configurations from all umbrella windows were combined into a generalized ensemble using MBAR. ^112^

#### Fine-tuning with thermodynamic reweighting

In the second stage, we further refine the force field against experimental measurements for a selected set of larger proteins (Table S7-S9), where constructing fully converged atomistic ensembles remains challenging. This step follows a top-down strategy, in which model parameters are adjusted to reproduce experimentally observed ensemble-averaged properties. Here, we use the *R_g_* as the target observable. The optimization is carried out using a thermodynamic reweighting framework, ^81^ combined with regularization based on effective sample size ^82^ to ensure stable and efficient parameter updates without additional simulations. This scheme allows estimation of how small perturbations in the energy function parameters affect an observable, i.e., *R_g_*, using reference ensembles that only need to be constructed once.

### Molecular dynamics simulation details

MOFF2 was implemented in OpenABC, ^36^ a Python-based simulation package built on OpenMM for GPU-accelerated molecular dynamics simulations of coarse-grained biomolecular systems.

#### Single-chain simulations

Simulations of individual proteins were performed in the NVT ensemble using the Langevin middle integrator, ^113^ with a friction coefficient of 1 ps^−1^ and a timestep of 10 fs. Each system was energy-minimized and equilibrated for 5 × 10^5^ steps before production. Production simulations were run for 2 × 10^8^ steps, corresponding to 2 μs, in a cubic box with a side length of 1000 nm. Coordinates were saved every 5 × 10^3^ steps, yielding 4 × 10^4^ frames per trajectory. Simulation temperatures and ionic strengths were matched to the corresponding experimental conditions when available, as summarized in Table S6-S10. For OP and MDP MOFF2 testing simulations without experimental conditions, simulations were performed at 298 K and 150 mM ionic strength, as summarized in Tables S11-S12.

#### Condensate simulations

For A1-LCD phase-separation simulations, the initial multi-chain configurations were generated by randomly inserting 100 copies of each A1-LCD variant into a rectangular simulation box of size 25 × 25 × 300 nm^3^. Systems were first compressed under NPT conditions using a Monte Carlo barostat ^114^ at 1 bar and the Langevin middle integrator with a 10 fs timestep. The NPT compression was performed for 8.0 × 10^4^ steps, and the resulting configuration was used to initialize the slab simulations. After compression, the barostat was removed and the simulation box was reset to 25 × 25 × 300 nm^3^. Slab simulations were performed at 150 mM ionic strength and at the temperature matched for experimental values (Table S13). Production simulations were performed for 2.0 *μ*s and configurations were saved every 1 × 10^5^ steps. The trajectories were recentered and aligned along the slab normal prior to calculating concentration profiles and saturation concentrations.

The saturation concentration (*C*_sat_) was estimated from slab simulations using the density of the dilute phase. For each A1-LCD variant, the first 1 *μ*s of the trajectory was discarded as equilibration. A one-dimensional *z*-density profile was computed from the retained frames and smoothed with a moving-average window. The bin with the maximum smoothed density was assigned as the dense-phase center. A dense-core region was then defined using neighboring bins around this maximum, with an additional 5 nm margin on both sides (red dashed line in Figure 4B). Protein chains outside this dense-core region were used to estimate the dilute-phase concentration.

### Analysis details.

#### UMAP sequence analysis.

UMAP ^83^ was used to visualize protein sequence-composition space across training, fine-tuning, and test sets. Each protein was represented by a 20-dimensional amino-acid composition vector containing the fractional abundance of each residue type. UMAP was then applied to the combined composition matrix using Euclidean distance, with *n*_neighbors_ = 20, min dist = 0.1.

#### PCA analysis of MOFF2 interactions.

To interpret the residue-level chemical information encoded by MOFF2, we analyzed the learned interaction components using principal component analysis (PCA). ^115,116^ PCA was applied separately to each MOFF2 component, including the AH interaction matrix λ_AH_, the first-solvation-shell Gaussian matrix *G*_FSS_, and the density-dependent many-body potential *V*_mb_ (Figures S6-S7).

For matrix-valued components, including λ_AH_, *G*_FSS_, each amino acid was represented by its interaction profile against all 20 amino acids, yielding a 20-dimensional vector per amino acid. For the density-dependent many-body term, each amino acid is represented by the energy values on a uniform grid of 500 local-density values between *ρ*_min_ = 0 and *ρ*_max_ = 15.

Before PCA, each profile matrix was standardized feature-wise using *z*-score normalization. The resulting PC scores provide compact amino-acid-level representations of the dominant chemical trends encoded by each learned component. In particular, PC1 was used as a one-dimensional residue profile for comparison with published hydrophobicity, solvation, and residue-interaction scales.

For reference comparisons, we included the Miyazawa–Jernigan model, ^102^ the HPS–Urry scale, ^25^ and 21 additional published residue-level scales. ^100,103,104,117-131^ Reference scales, except for Miyazawa–Jernigan model, were provided as one-dimensional amino-acid profiles and standardized using *z*-score normalization for comparisons with the MOFF2 PC1 profiles. The Miyazawa–Jernigan model was first analyzed using the same PCA procedure applied to the MOFF2 matrix-valued components. Its resulting PC1 amino-acid profiles were then used for comparison.

**Relative root-mean-square error (rRMSE).** rRMSE was used to quantify the prediction error relative to the reference value for each protein,

$$\text{rRMSE}=\sqrt{\frac{1}{N}\sum_{i=1}^{N}{\left(\frac{y_{i}-x_{i}}{x_{i}}\right)}^{2}}.$$

Here, $x_{i}$ and $y_{i}$ are the reference and predicted values for protein i, respectively, and N is the number of proteins in the corresponding evaluation set.

**Mean signed deviation** (Δ). Δ was used to quantify systematic bias,

$$\Delta =\frac{1}{N}\sum_{i=1}^{N}(y_{i}-x_{i}).$$

Here, $x_{i}$ and $y_{i}$ are the reference and predicted values for protein i, respectively, and N is the number of proteins in the corresponding evaluation set. Positive ∆ indicates that the model overestimates the reference values on average, whereas negative ∆ indicates underestimation.

## Supplementary Material

Supplement 1
